# Pseudotumor cerebri syndrome in a child with Alagille syndrome: intracranial pressure dynamics and treatment outcome after ventriculoperitoneal shunting

**DOI:** 10.1007/s00381-021-05043-9

**Published:** 2021-02-08

**Authors:** Manolis Polemikos, Elvis J. Hermann, Hans E. Heissler, Hans Hartmann, Joachim K. Krauss

**Affiliations:** 1 Department of Neurosurgery, Hannover Medical School, MHH, Carl-Neuberg-Str. 1, DE-30625 Hannover, Germany; 2 Clinic for Paediatric Nephrology, Hepatology and Metabolic Disorders, Hannover Medical School, Hannover, Germany

**Keywords:** Idiopathic intracranial hypertension, Pseudotumor cerebri, Alagille syndrome, Ventriculoperitoneal shunting

## Abstract

Alagille syndrome (AS) is a rare multisystem disease of the liver, heart, eyes, face, skeleton, kidneys, and vascular system. The occurrence of pseudotumor cerebri syndrome (PTCS) in patients with AS has been reported only exceptionally. Owning to its rarity and a mostly atypical presentation, the diagnosis and natural history of affected patients remain uncertain. We report an atypical case of PTCS in a 4-year-old boy with a known history of AS who presented with bilateral papilledema (PE) on a routine ophthalmological examination. Visual findings deteriorated after treatment with acetazolamide. Continuous intracranial pressure (ICP) monitoring was then utilized to investigate ICP dynamics. Successful treatment with resolution of PE was achieved after ventriculoperitoneal shunting but relapsed due to growth-related dislocation of the ventricular catheter. This report brings new insights into the ICP dynamics and the resulting treatment in this possibly underdiagnosed subgroup of PTCS patients. It also demonstrates that ventriculoperitoneal shunting can provide long-term improvement of symptoms for more than 10 years.

Alagille syndrome (AS) or arteriohepatic dysplasia is a rare (1:30,000) autosomal-dominant inherited multisystem disorder [15]. It primarily affects the liver, heart, eyes, face, skeleton, kidneys, and vascular system, whereas its expressivity (phenotypic severity) is highly variable, ranging from no apparent clinical involvement to severe disease that requires liver transplantation [1, 8, 16, 19].

Pseudotumor cerebri syndrome (PTCS) is a disorder characterized by elevated intracranial pressure (ICP) with normal cerebrospinal fluid (CSF) composition in the absence of hydrocephalus, mass lesions, or structural abnormalities. Common presenting signs and symptoms of elevated ICP include papilledema (PE), visual disturbances, and headache. PTCS can be differentiated in a primary form of unknown etiology, which is also termed idiopathic intracranial hypertension (IIH), and a secondary form in which intracranial hypertension results from a medical condition or an exogenous agent [2, 11, 12, 24].

The occurrence of PTCS in AS is exceedingly rare [9, 10, 20, 21]. Thus far ICP monitoring has not been reported in this subgroup of PTCS. Furthermore, owing to their rarity, the natural history and treatment of PTCS in AS patients remain unclear. Here, we present the clinical findings, ICP dynamics, and treatment outcome in a 4-yead-old boy with AS and associated PTCS. Furthermore, previous reports are being reviewed and the possible pathophysiology is discussed.

## Case report

### History and examination

This 4-year-old boy had a known history of AS. He was born full term at 40 weeks’ gestation by Cesarean section secondary to cephalopelvic disproportion following an uncomplicated pregnancy. At 28 days of age, he underwent a Kasai procedure for biliary atresia. Due to progression of his liver disease, a liver transplantation was performed at 11 months of age after AS had been diagnosed. At 4 years of age, bilateral PE was detected in a routine ophthalmological examination.

He was then referred to the neuropediatric department for further evaluation. On clinical examination, he had a normal blood pressure, his weight was 16.5 kg, and his height 96 cm. Neuropediatric examination was normal. Laboratory studies including renal and liver function parameters were normal. An echocardiogram revealed no cardiovascular abnormalities.

Medication consisted of immunosuppressive therapy with ciclosporine (70 mg/day), mycophenolate mofetil (400 mg/day), and prednisolone (1 mg/day). The cyclosporine level was within normal range.

An MRI scan was performed in which hydrocephalus, mass effect, or structural lesion was ruled out. Subsequently, a lumbar puncture in propofol sedation was achieved revealing a recumbent opening pressure of 48 cm H_2_O. Examination of the CSF including bacteriological and viral panels showed no abnormalities. Consequently acetazolamide treatment was initiated at a 2 × 125 mg dosage. After 6 weeks, the PE persisted and also deterioration of the visual acuity was documented. In a repeat LP, the opening pressure was still elevated at 34 cm H_2_O. Repeat neuroimaging assessment remained unremarkable.

Given the progressive visual loss and the PE being refractory to medical treatment, the patient was referred to the Department of Neurosurgery for further evaluation and measurement of ICP dynamics.

### Intracranial pressure monitoring

**Fig. 1:**
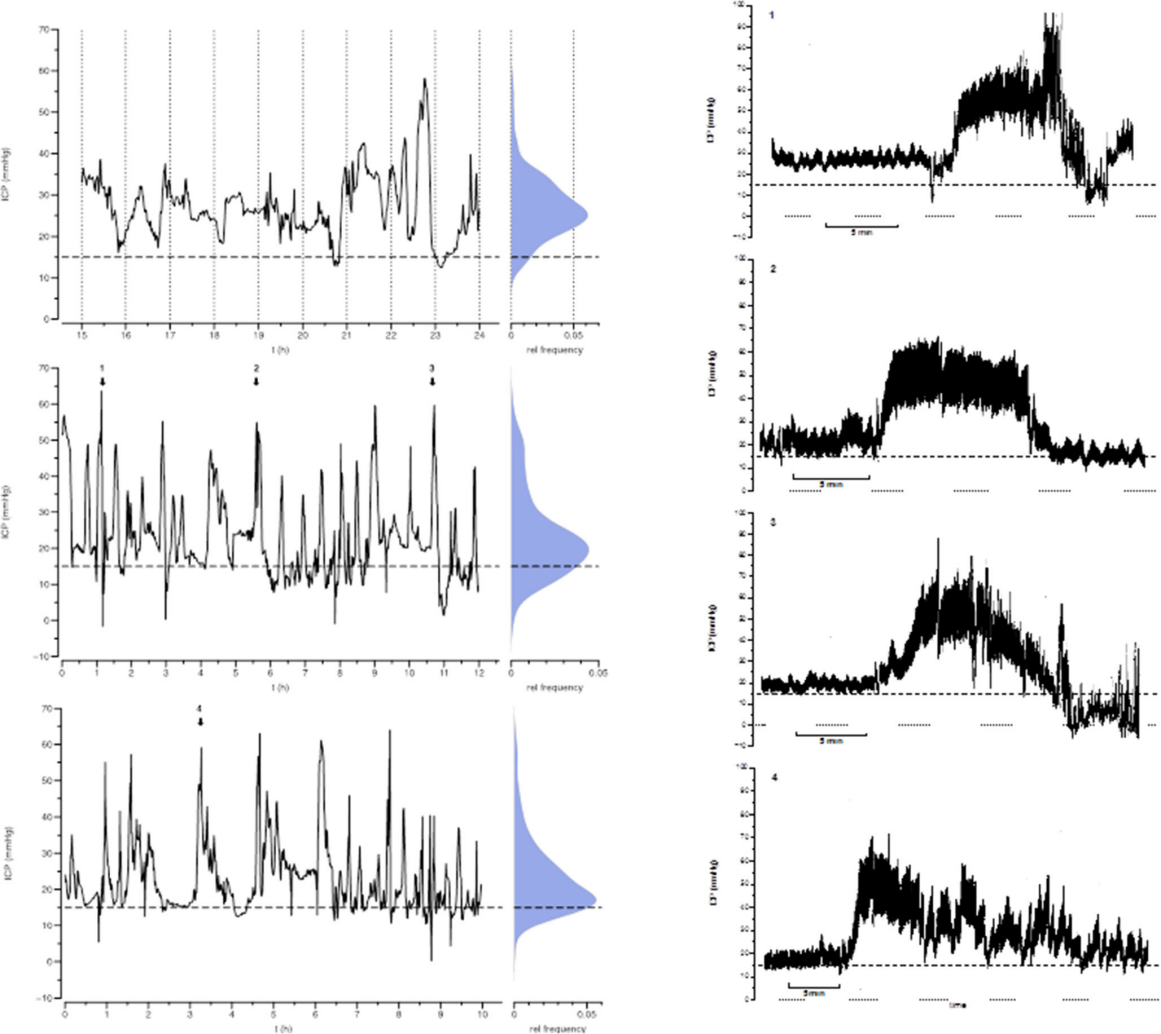
Intracranial pressure dynamics in a 4-year-old boy with PTCS in Alagille syndrome. The traces show distinctive pathological pressure transients of high amplitude. Data are outlined as traces indicating mean pressure, and as smoothed histograms in a vertical layout. The upper limit of physiological ICP was set at 15 mmHg and is presented as dashed line. On the right side four samples of ICP transients (1–4) demonstrate the complex dynamics of the elevated ICP. Dotted line: zero pressure; brackets: time intervals of 5 min

An epidural sensor (Neurodur®, Raumedic, Münchberg, Germany) was implanted over the right frontal area via a precoronal burrhole, and the ICP was monitored for 2 days. ICP monitoring demonstrated massive dynamic ICP changes over time with markedly increased ICP values most of the time (Fig. 1). The decomposition of pressure traces revealed pressure waves with amplitudes reaching values up to 60 mmHg for several minutes. B-waves dominated ICP dynamics superposing the slower ICP fluctuations in time. Additionally, a relatively high number of A-wave pressure transients were scattered in the traces. The majority of ICP values were measured to be within the range of 15 to 70 mmHg (83%).

### Shunt surgery and postoperative course

Subsequent to ICP monitoring, a ventriculoperitoneal shunt was implanted using an optic-guided neuronavigation system (Medtronic, Minneapolis, MN, USA). A proGAV® valve with an integrated shuntassistant® (Aesculap-Miethke, Tuttlingen, Germany) was implanted at an opening pressure of 10 cm H_2_0 [25]. The CT scan on the first postoperative day demonstrated an accurately placed ventricular catheter, and the patient was discharged on the seventh postoperative day.

One month after discharge, PE had completely resolved. Further follow-ups were unremarkable until a growth-related dislocation of the ventricular catheter was noted 3.5 years after the shunting procedure. An ophthalmological examination showed relapse of bilateral PE. The ventricular catheter was revised and the immediate postoperative course was uneventful. One month following discharge, the shunt system was explanted due to an infection with *Staphylococcus aureus* and an external ventricular drain was placed. Antibiotics were administrated intravenously for 3 weeks. When serial CSF bacterial cultures were negative, a new ventriculoperitoneal shunt was inserted guided by electromagnetic neuronavigation (AxiEM) [14]. The valve was set at an opening pressure of 6 cm H_2_O. Postoperative CT confirmed an accurately placed ventricular catheter. At long-term follow-up (12 years), there was no recurrence and both ophthalmological and neurological findings were unremarkable. The opening pressure has not been adjusted during the follow-up period.

## Discussion

The natural course of AS is associated with high morbidity and mortality if not being diagnosed timely and treated appropriately [16]. AS is associated with a plethora of ophthalmological abnormalities, predominately posterior embryotoxon, optic disc drusen, angulated retinal vessels, and pigmentary retinopathy [17]. In the majority of patients, normal vision can be secured [18]. PE is the most common sign of elevated ICP in PCTS, which can lead to progressive visual loss, when left untreated [26]. Although PE is not associated commonly with AS, concomitant congenital optic disc anomalies can be misinterpreted as PE and consequently result in misdiagnosis and unnecessary treatment [5, 17].

The typical patient with primary PTCS or IIH is a young obese woman in childbearing age [12, 29]. Establishing the diagnosis of primary PCTS in a typical patient is mostly a straightforward process based on the 2013 revision of the Friedman & Jacobson criteria [12]. These require that in the presence of PE the opening pressure measured at lumbar puncture is elevated, the CSF composition is normal, there is no evidence of neurological deficits except for cranial nerve abnormalities, and there are no pathological findings in MRI with and without gadolinium [5, 11, 12]. In contrast, the clinical profile of pediatric PCTS differs widely. Pediatric patients frequently remain asymptomatic but PE is incidentally diagnosed in up to 30% during a routine eye examination [4, 13, 27, 28]. Correct diagnosis in pediatric patients is challenging. Opposite to adults, in children gender distribution is equal and weight is not a contributing factor for disease development. It has been estimated that up to 30% of children with PTCS and a normal body weight harbor a secondary etiology; therefore, appropriate identification of causative factors and conditions is essential prior further treatment [3, 27].

The optimal treatment of PTCS in children remains debatable. The main stay of PTCS medical treatment in adults is acetazolamide. Surgical treatment (CSF shunting procedures, optic nerve sheath fenestration, and venous stenting) is indicated when visual loss is progressive and/or symptoms are intractable despite maximal medical management [14, 23, 28].

Although continuous ICP-monitoring is not routinely performed for diagnosing PTCS, it has been shown that its application is a helpful adjunct especially in atypical cases and when surgical treatment is considered [23, 30]. Especially in pediatric patients, it can provide an accurate measurement of ICP, which is commonly overestimated through a lumbar puncture [6]. An increased baseline ICP, large ICP fluctuations, and the presence of ICP-oscillations (A- and B-waves) during recordings further can confirm the diagnosis [23, 30].

The occurrence of PCTS in patients with AS is exceedingly rare with only few patients having been reported thus far (Table 1). Identifying true risk factors is important for establishing the diagnosis of PCTS and also for understanding the pathophysiology of the disorder [7].

**Table 1:** Summary of patients with PTCS and Alagille syndrome

Authors and year	Age at presentation/sex	Clinical presentation	Opening pressure/cm H20	Time between LT and PTCS development/immunosuppression	Vitamin A level/supplementation	Therapy	Outcome	Follow-up duration (years)
Emerick et al., 2005	3 yrs./M	symptomatic, PE	Raised/24	Not transplanted	ns/ns	Acetazolamide and dexamethasone	Full recovery	ns
Narula et al., 2006	6 yrs./NS	symptomatic, PE	Raised/ns	4 yrs./tacrolimus and steroids	Normal/no	Acetazolamide and prednisolone, LPS after 15 mo	PE resolved, normal vision	ns
	25 mo/NS	asymptomatic PE	Raised/ns	16 mo/tacrolimus (changed from cyclosporine due to early rejection)	Elevated before PTCS development, normal after PTCS development/ns	Furosemide	Normal CSF pressure and vision	ns
	20 mo/NS	Asymptomatic PE	Raised/ns	Not transplanted	Normal/no	Repeated LP (3×)	Normal development and vision	ns
Ertekin et al., 2010	5 yrs./M	Symptomatic	Raised/29	Not transplanted	Elevated/yes, less than recommended levels	Repeated LPs, acetazolamide	Symptom free	ns
Mouzaki et al., 2010	25 mo/F	Asymptomatic PE	Raised/37	Not transplanted	Elevated/no	Acetazolamide	Improvement of PE	0.5
	13 mo/M	Asymptomatic PE	Raised/35	Not transplanted	Elevated/no	Acetazolamide	Improvement of PE	ns
Polemikos et al., 2020	4 yrs./M	Asymptomatic PE	Raised/48	3 yrs./cyclosporine, steroids, mucophenolat mofetil	Normal/no	Acetazolamide, VPS after 6 weeks	Complete resolution of PE	12

*F*, female; *M*, male; *LP*, lumbar puncture; *LPS*, lumboperitoneal shunt; *LT*, liver transplantation; *ns*, not stated; *PE*, papilledema; *VPS*, ventriculoperitoneal shunt; *PTCS*, pseudotumor cerebri syndrome

Both hypervitaminosis and hypovitaminosis A are risk factors for PCTS, and may be relevant in AS since most patients have a fat-soluble vitamin deficiency of variable degree which necessitates supplementation [15]. Previous reports have considered elevated vitamin A levels as causative agent of PCTS in AS, two of them in the absence of vitamin A supplementation [21] and one while on receiving vitamin A and D in less than recommended levels [10].

From a pathophysiological point of view, it has been hypothesized that an increased production of CSF or an increased resistance to CSF outflow is the underlying mechanisms for PTCS, whereas most evidence supports the latter, possibly due to a field effect involving epithelial membranes [20]. Sheldon et al. proposed a neuroendocrine pathomechanism for pediatric PTCS. They hypothesized that in PTCS hormonal and metabolic factors regulate CSF production and CSF absorption ultimately leading to elevated ICP [24]. Furthermore, genetic or epigenetic factors appear to be relevant for developing PCTS, based on previously reported familial cases of PTCS [23].

In 89% of cases, AS is caused by mutations/deletions in the Notch signaling pathway ligand gene, JAGGED1, or the gene for its receptor, NOTCH2 [16]. The Notch signaling pathway has an important role in vascular development. Disruption of this pathway results in abnormal vascular development and signaling, which may also contribute to the development of IIH [15, 22]. Mouzaki et al. speculated that abnormalities in the microvasculature of the choroid plexus in AS patients could lead to abnormal CSF production or absorption, which in turn would cause intracranial hypertension [21].

The occurrence of PTCS in AS patients after liver transplantation has been previously described in 2 cases by Narula et al. [22]. While these patients were on tacrolimus, our patient was receiving cyclosporine, which has been related to the development raised ICP after renal, bone marrow, and heart transplantation. Nevertheless, based on the occurrence of intracranial hypertension in AS patients without liver transplant, it appears unlikely that the immunosuppressive medication solely resulted in PCTS, although it could have been a contributing factor [22].

Due to its rarity, knowledge about treatment of PTCS in AS remains very limited. This is reflected by the few previously reported cases as outlined in Table 1. While medical treatment with acetazolamide, furosemide, and steroids was effective, there is only very little information on long-term efficacy. We here show that ventriculoperitoneal shunting may provide long-term relief and prevent recurrent PE. Relapse of PE due to growth-related dislocation of the ventricular catheter reinforces the need of ophthalmological and radiological follow-up examinations.

PTCS in AS is likely to be underdiagnosed because ophthalmological examinations are not performed routinely after initial diagnostic workup and due to the fact that the majority of patients remain asymptomatic [22].

## Conclusions

Our report emphasizes the need for careful evaluation of PCTS in pediatric AS. Through the utility of continuous ICP monitoring, we offer new insights regarding ICP dynamics of this population. We also demonstrate that CSF shunting may provide reliable relief of symptoms on long-term follow-up. Our findings along with other reported cases suggest that a genetic predisposition makes AS patients susceptible for developing PCTS, whereas associated medical conditions and exogenous factors can trigger or exacerbate intracranial hypertension. The differentiation of PTCS in AS as primary or secondary PCTS is a subject deserving further discussion with findings thus far being supportive for the first.

## Data Availability

Not applicable.

## Abbreviations

AS: Alagille syndrome
CSF: Cerebrospinal fluid
ICP: Intracranial pressure
IIH: Idiopathic intracranial hypertension
PE: Papilledema
PTCS: Pseudotumor cerebri syndrome
